# Supplementary material for: On the engineering of higher-order Van Hove singularities in two dimensions
Source: Nat Commun. 2024 Nov 4;15:9521. doi: 10.1038/s41467-024-53650-2 (PMC11535232; doi:10.1038/s41467-024-53650-2)
Supplement: Supplementary file 1 — Supplementary Information [file 41467_2024_53650_MOESM1_ESM.pdf]

Supplementary Information for ‘On the engineering of higher-order Van Hove singularities in two dimensions’

Anirudh Chandrasekaran,[1,*] Luke C. Rhodes,[2] Edgar Abarca Morales,[2,3] Carolina A. Marques,[2,4] Phil D.C. King,[2] Peter Wahl,[2,5] and Joseph J. Betouras[1,†]

[1]*Department of Physics and Centre for the Science of Materials, Loughborough University, Loughborough LE11 3TU, United Kingdom*

[2]*SUPA, School of Physics and Astronomy, University of St Andrews, North Haugh, St Andrews, KY16 9SS, United Kingdom*

[3]*Max Planck Institute for Chemical Physics of Solids, Noethnitzer Strasse 40, 01187 Dresden, Germany*

[4]*Physik-Institut, Universität Zürich, Winterthurerstrasse 190, CH-8057 Zürich, Switzerland*

[5]*Physikalisches Institut, Universität Bonn, Nussallee 12, 53115 Bonn, Germany*

(Dated: October 15, 2024)

[*]Corresponding author: A.Chandrasekaran@lboro.ac.uk

[†]Corresponding author: J.Betouras@lboro.ac.uk

Supplementary Note 1: Crossover from log to power-law in the DoS in the vicinity of a HOVHs

As we tune θ, we will find that the series expansion at the $\overline{\mathrm{M}}$ point takes the form $\alpha\, k_x^2 + \beta k_y^2 + \gamma\, k_x^4 + \mu\, k_x^2 k_y^2 + \nu\, k_y^4$ along with a $\pi/2$ rotated copy obtained by applying $(k_x, k_y) \to (-k_y, k_x)$.

Since the pair of symmetry related bands are conjoined together, we need a strategy to isolate a particular band of interest out of the two. Now the series expansion for θ in the range $[7^\circ, 12^\circ]$ indicates that $\nu \ll \gamma$ for one band and $\nu \gg \gamma$ for the other one. For example, at $\theta = 9^\circ$, the unrenormalized, SOC-free model has the following series expansion at the $\overline{\mathrm{M}}$ point: $-0.09 - 0.029\, k_x^2 + 0.063\, k_y^2 - 0.4\, k_x^4 + 0.027\, k_y^2 k_x^2 - 0.04\, k_y^4$ (along with its rotated counterpart. Check the Mathematica notebook '4_Log_to_Power_law.nb' in the repository [1] for more details). We can see that the coefficient of the k_x^4 term is about ten times larger than the coefficient of the k_y^4 term. This feature persists for all θ in that range. Therefore, to be consistent, let us choose the band with $\nu < \gamma$ for further analysis. The DoS integral is given by

$$g(\epsilon) = \int d^2k\, \delta\left(\alpha\, k_x^2 + \beta\, k_y^2 + \gamma\, k_x^4 + \mu\, k_x^2 k_y^2 + \nu\, k_y^4 - \epsilon\right),$$

where we have ignore the pre-factors and have assumed that the integration is performed over an appropriate region of the k-space. To infer the approximate low energy scaling behaviour of the DoS (particularly for a HOVHs), one normally extends the integrals over the entire (k_x, k_y)-plane (picking up a finite error in the process) and re-scales the k_x and k_y in an appropriate fashion. When α is "small" (to be qualified below) we will re-scale $(k_x, k_y) \to (|\epsilon|^{1/4} k_x, |\epsilon|^{1/2} k_y)$ to obtain after simplification

$$g(\epsilon) \sim \int d^2k\ |\epsilon|^{\frac{1}{4}+\frac{1}{2}}\, \delta\left(\alpha\, |\epsilon|^{1/2}\, k_x^2 + \beta\, |\epsilon|\, k_y^2 + \gamma\, |\epsilon|\, k_x^4 + \mu\, |\epsilon|^{3/2}\, k_x^2 k_y^2 + \nu\, |\epsilon|^2\, k_y^4 - \epsilon\right)$$

Assume that $\epsilon > 0$ (the arguments can be applied with a slight modification for $\epsilon < 0$). Then, using the scaling property of the delta function we obtain

$$g(\epsilon) \sim \int d^2k\ \epsilon^{\frac{1}{4}+\frac{1}{2}-1}\, \delta\left(\frac{\alpha\, k_x^2}{\sqrt{\epsilon}} + \beta\, k_y^2 + \gamma\, k_x^4 + \mu\, \sqrt{\epsilon}\, k_x^2 k_y^2 + \nu\, \epsilon\, k_y^4 - 1\right)$$

Since $[\epsilon] = \mathcal{E}$, $[\alpha] = \mathcal{E}.l^2$ and $[\gamma] = \mathcal{E}.l^4$, we have $[\alpha^2\epsilon^{-1}] = \mathcal{E}.l^4 = [\gamma]$, where $\mathcal{E}$ and l are respectively energy and length units. Thus, $\alpha^2\epsilon^{-1}$ and γ are comparable since they have the same dimension. In fact, for energies satisfying $\alpha^2\epsilon^{-1} \ll \gamma$, we can ignore the k_x^2 term in comparison to the k_x^4 in the DoS integral by further rescaling $k_x \to k_x/\gamma^{1/4}$. (This is what we meant by α being small earlier). For sufficiently small energies, the terms containing $\epsilon^{1/2}$ and ϵ can also be ignored so that the final DoS integral scales approximately as

$$g(\epsilon) \sim \epsilon^{-1/4} \int d^2k\, \delta\left(\beta\, k_y^2 + \gamma\, k_x^4 + \nu\, k_y^4 - 1\right),$$

where the integral has become ϵ independent due to the approximation. We proceed similarly for $\epsilon < 0$, obtaining for $\alpha^2|\epsilon^{-1}| \ll \gamma$

$$g(\epsilon) \sim |\epsilon|^{-1/4} \int d^2k\, \delta\left(\beta\, k_y^2 + \gamma\, k_x^4 + \nu\, k_y^4 + 1\right),$$

Thus the crossover from log to power law is controlled by

$$|\epsilon| \gg \frac{\alpha^2}{\gamma}.$$

That is, for energies well above this scale, the quartic term dominates over the quadratic term in the DoS integral, giving an approximate power law behaviour while for energies well below this scale, the quadratic term dominates, giving a logarithmically diverging DoS.

Supplementary Note 2: An alternate tuning scheme - staggered chemical potential

Starting from the Wannierized tight-binding model for Sr_2RuO_4 we can also engineer a four-fold symmetric X_9 singularity. This has a more strongly divergent DoS exponent of -1/2 as compared to the A_3 singularity with a DoS exponent of -1/4 [2]. Recall that in the θ-interpolated model, we had a pair of d_{xy} bands that become degenerate at the $\overline{\mathrm{M}}$ point and jointly satisfy the four-fold rotation symmetry. We managed to tune these bands to a pair of cusp (A_3) singularities (with canonical forms $k_x^4 - k_y^2$ and $k_x^2 - k_y^4$). The

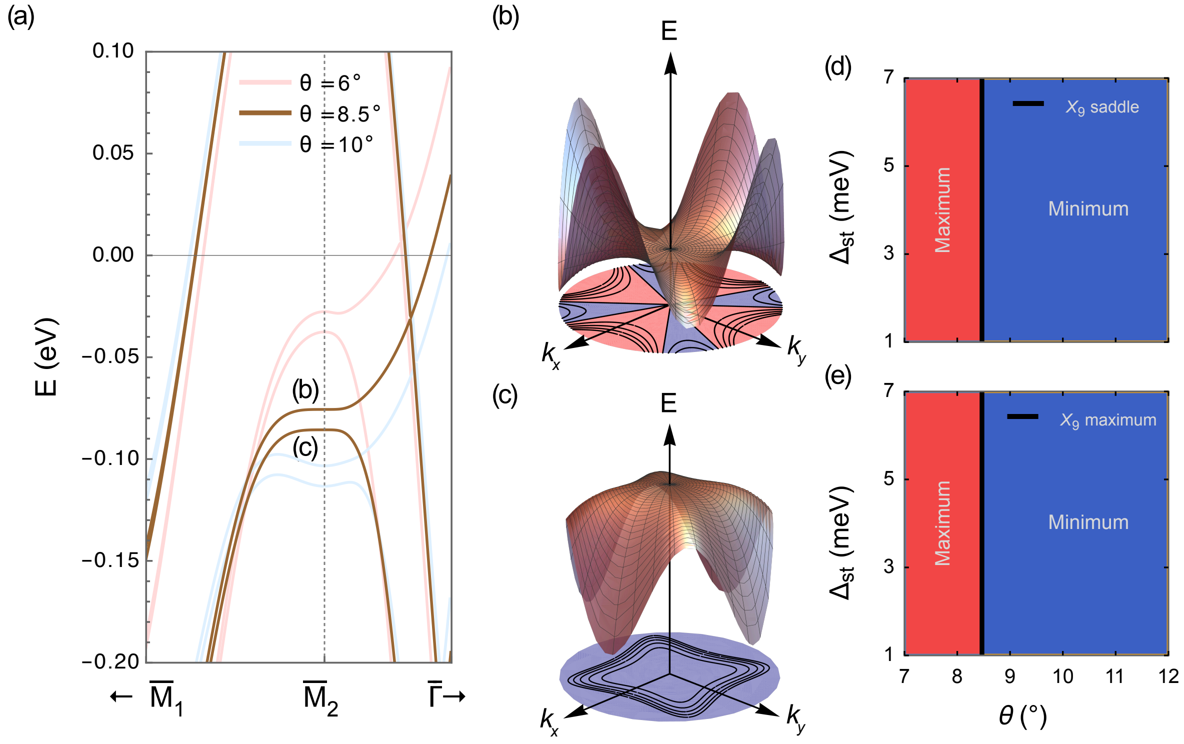

Supplementary Figure 1: Tuning to an X_9 singularity. (a) A staggered chemical potential Δ_{stag}, when added to the vanilla model for Sr_2RuO_4, makes the two Ru atoms nonequivalent. This lifts the degeneracy of the d_{xy} bands at the $\overline{\mathrm{M}}$ point while still preserving the four-fold rotation symmetry. When the RuO octahedral angle θ is tuned, the bands evolve from a pair of maxima (light red bands) to a pair of minima (light blue bands), passing through a pair of X_9 singularities at $\theta \approx 8.5°$ (the brown bands). These X_9 HOVHs take the form of a saddle (b) and a higher order maximum (c), both having a divergent DoS exponent of -1/2. In (d) and (e) we depict the phase diagrams for the bands (b) and (c) respectively. These track the evolution of the nature of the critical point as θ and Δ_{stag} are tuned.

degeneracy at the $\overline{\mathrm{M}}$ is guaranteed by the lattice reflection symmetry that makes the two Ru atoms equivalent. By adding a staggered chemical potential Δ_{stag} that makes them nonequivalent, we can break the degeneracy at the $\overline{\mathrm{M}}$ point while still preserving the four-

fold rotation symmetry. In fact, we notice that the two d_{xy} bands can be tuned to host a pair of X_9 singularities at $\theta \approx 8.5^\circ$, with one band hosting a four-fold saddle and the other hosting a higher order maximum (see Supplementary Figure 1 and the Mathematica notebook '3_M_point_staggered_chemical_potential.nb' in the GitHub repository [1]). Both have the canonical form $k_x^4 + c\,k_x^2 k_y^2 + k_y^4$, with the sign and magnitude of c determining whether the singularity is a saddle, maximum or minimum.

Supplementary References

[1] Anirudh Chandrasekaran. Tuning higher order singularities in Sr_2RuO_4, June 2024. URL `https://github.com/anirudhc-git/VHS_Sr2RuO4`.

[2] A. Chandrasekaran, A. Shtyk, J. J. Betouras, and C. Chamon. Catastrophe theory classification of Fermi surface topological transitions in two dimensions. *Phys. Rev. Research*, 2 (1):013355, March 2020. ISSN 2643-1564. doi: 10.1103/PhysRevResearch.2.013355. URL `https://link.aps.org/doi/10.1103/PhysRevResearch.2.013355`.
